# Retraction: Preterm birth is not associated with asymptomatic/mild SARS-CoV-2 infection *per se*: Pre-pregnancy state is what matters

**DOI:** 10.1371/journal.pone.0339112

**Published:** 2025-12-18

**Authors:** The PLOS One Editors

The *PLOS One* Editors retract this article [1] because it was identified as one of a series of submissions for which we have concerns about compromised peer review and compliance with PLOS policy on Manipulation of the Publication Process.

SC, JC, FB, MC, and IS did not agree with the retraction. ARC responded but expressed neither agreement nor disagreement with the editorial decision. FG, MP, VG, GDP, and CB either did not respond directly or could not be reached.
